# Role of phenolics from *Spondias pinnata* bark in amelioration of iron overload induced hepatic damage in Swiss albino mice

**DOI:** 10.1186/s40360-016-0077-6

**Published:** 2016-07-26

**Authors:** Dipankar Chaudhuri, Nikhil Baban Ghate, Sourav Panja, Nripendranath Mandal

**Affiliations:** Division of Molecular Medicine, Bose Institute, P 1/12, C. I. T. Road, Scheme – VIIM, Kolkata, 700054 West Bengal India

**Keywords:** Hemosiderosis, Oxidative stress, Antioxidant enzymes, Lipid peroxidation, Liver fibrosis

## Abstract

**Background:**

Crude *Spondias pinnata* bark extract was previously assessed for its antioxidant, anticancer and iron chelating potentials. The isolated compounds gallic acid (GA) and methyl gallate (MG) were evaluated for their curative potential against iron overload-induced liver fibrosis and hepatocellular damage.

**Methods:**

In vitro iron chelation property and in vivo ameliorating potential from iron overload induced liver toxicity of GA and MG was assessed by different biochemical assays and histopathological studies.

**Results:**

MG and GA demonstrated excellent reducing power activities but iron chelation potential of MG is better than GA. Oral MG treatment in mice displayed excellent efficacy (better than GA) to significantly restore the levels of liver antioxidants, serum markers and cellular reactive oxygen species in a dose-dependent fashion. Apart from these, MG exceptionally prevented lipid peroxidation and protein oxidation whereas GA demonstrated better activity to reduce collagen content, thereby strengthening its position as an efficient drug against hepatic damage/fibrosis, which was further supported by histopathological studies. Alongside, MG efficiently eliminated the cause of liver damage, i.e., excess iron, by chelating free iron and reducing the ferritin-bound iron.

**Conclusions:**

The present study confirmed the curative effect of GA and MG against iron overload hepatic damage via their potent antioxidant and iron-chelating potential.

**Electronic supplementary material:**

The online version of this article (doi:10.1186/s40360-016-0077-6) contains supplementary material, which is available to authorized users.

## Background

An imbalance in pro-oxidant/antioxidant homeostasis eventually gives rise to the physical condition known as “oxidative stress”. An irregular surge in oxidants such as reactive species of oxygen (ROS) and nitrogen (RNS) is generated endogenously through mitochondrial respiration as well as through exogenous oxidizing agents including ionizing radiation, heavy metals, and hypoxia. These oxidants come in contact with different biological molecules such as proteins, lipids and DNA, thereby disrupting them and causing maladies such as cancer, atherosclerosis, cardiovascular disease, liver injury, aging and inflammatory diseases [1]. Previous research has indicated that redox-active metals such as iron also play a major role in ROS over production, mainly through highly reactive hydroxyl radicals by undergoing redox cycling [2]. Conversely, liver executes the normal metabolic balance of the body as well as biotransformation, detoxification and excretion of several toxic by-products. Increasing evidence has indicated that oxidative stress and its associated damage designate a common association between different forms of chronic liver injuries and hepatic fibrosis [3] due to iron overload [4]. Thus, any substance that is capable of providing protection against metal toxicity by trapping free radicals or by chelating metal ions that would consequently terminate the chain reaction of free radicals acts as an antioxidant [5]. In addition to endogenous antioxidant systems, frequent consumption of food that is rich in natural antioxidants also exhibits increased resistance to oxidative stress by altering the redox environment and is associated with a lower risk of many oxidative stress-related diseases.

Several phenolics and flavonoids from natural resources effectively scavenge different free radicals through their relevant iron chelating capabilities. Previous reports suggested that these types of compounds could also be used to treat iron-induced liver toxicity [6]. Crude *Spondias pinnata* (Linn. f.) Kurz (Fam. Anacardiaceae) extract was previously studied for its in vitro and in vivo antioxidant and iron chelating potential, which also demonstrated the presence of significant amounts of phenolic and flavonoid compounds [7, 8]. These previous studies prompted us to isolate phenolic compounds from *Spondias pinnata* bark and evaluate their ameliorating effect on iron overload-induced hepatotoxicity and hepatic fibrosis in mice.

## Methods

### Reagents

Bathophenanthroline sulfonate disodium salt, 5,5′-dithiobis-2-nitrobenzoic acid (DTNB) and N-(1-Naphthyl) ethylenediamine dihydrochloride (NED) were procured from Sisco Research Laboratories Pvt. Ltd, India. 1-chloro-2,4-dinitrobenzene (CDNB), Dimethyl-4-aminobenzaldehyde, and N,N- dimethyl-4-nitrosoaniline ammonium iron (II) sulfate hexahydrate ((NH_4_)_2_Fe(SO_4_)_2,_ 6H_2_O) were obtained from Merck, Mumbai, India. Guanidine hydrochloride and Iron-dextran was purchased from Sigma-Aldrich, USA. Cipla Ltd., India provided Desirox (Generic name-Deferasirox). All of the other used reagents were of molecular biology grade and were obtained from reputable suppliers.

### Test animals

In vivo experiments were performed abiding by the guidelines of the Committee for the Purpose of Control and Supervision of Experiments on Animals (CPCSEA), Ministry of Environment and Forest, Govt. of India with due approval from the Institutional Animal Ethics Committee, Bose Institute (Registration. No. 95/1999/CPCSEA). Male Swiss albino mice (20 ± 2 g) were obtained from the Chittaranjan National Cancer Institute (CNCI), Kolkata, India and were acclimated under a constant 12 h light/dark cycle at 22 ± 2 °C. The animals were fed on general laboratory diet and water *ad libitum.* Experimental animals were taken care every 6 h during the treatment period and it was observed that there was no unwanted animal death. All surgeries were done using ethyl ether as anesthetic (by inhalation in a ethyl ether saturated chamber inside a fume hood), taking utmost care to reduce suffering.

### Plant material

*S. pinnata* bark was collected from the villages of Bankura district, West Bengal, India and the plant was authenticated by Dr. Jayaram Hazra, Director, Central Research Institute of Ayurveda (CRIA), Kolkata, India. The herbarium was submitted at CRIA, Kolkata with an accession no of CRHS 111/08.

### Extraction and isolation of phyto-compounds

Different compounds were isolated following the method of Chaudhuri, et al. [9]. Briefly, the *S. pinnata* stem bark was cut into small pieces, dried, ground into powder, and extracted with 70 % methanol and water. The lyophilized extract was re-extracted successively with hexane, chloroform, ethyl acetate and water. All of the fractions were concentrated in reduced pressure, and the ethyl acetate fraction was further purified through silica gel column chromatography. Dichloromethane and methanol elution yielded four compounds namely SPE1, SPE2, SPE3 and SPE4. Structures of the bioactive compounds were analysed using different spectroscopic methods such as EIMS (JEOL JMS-700, Germany), FT-IR spectra recorded in KBr (Perkin Elmer, USA) and different nuclear magnetic resonance (NMR) experiments including ^1^H and ^13^C with a Bruker-500 MHz NMR Spectrometer (Germany).

### In vitro reducing power and iron chelation potential of the isolated compounds

Fe^3+^-reducing power of the compounds was determined by a standard method [10] where ascorbic acid was used as standard. Various concentrations (0–1.0 mg/ml) of the compounds were tested and their absorbance was measured at 700 nm against an appropriate blank. The Fe^2+^ chelating ability was determined as described earlier, and the result was expressed as inhibition percentage [7]. Briefly, in HEPES buffer (20 m mol/l, pH 7.2) medium, test samples (0–120 μg/ml) and positive control EDTA (0–20 μg/ml) were separately added to a 12.5 μ mol/l ferrous sulfate solution and 75 μ mol/l ferrozine was added to start the reaction. After rapid vortexing the mixture was left at room temperature for 20 min and absorbance was taken at 562 nm (OD). The percentage of inhibition of ferrozine-Fe^2+^ complex formation was determined by the formula- % of inhibition = [(Control OD-Sample OD)/Control OD] × 100. All tests were performed six times.

### In vivo hepato-ameliorating activity

#### Experimental design and tissue preparation

In total, nine groups of mice were randomly prepared consisting of six mice per group. Among them, one group received normal saline only, which was labeled as a blank (B); however, other groups were intoxicated with five doses of 100 mg/kg b.w. iron-dextran (one dose every alternative days) by intraperitoneal injection (ip). Of the groups, one iron-dextran group (C) was treated orally with only saline, and other groups were treated orally with 2 mg/kg b.w. gallic acid (GA) (R2), 2 mg/kg b.w. methyl gallate (MG) (S2), 4 mg/kg b.w. GA (R4), 4 mg/kg b.w. MG (S4), 8 mg/kg b.w. GA (R8), 8 mg/kg b.w. MG (S8) test samples and 20 mg/kg b.w. desirox (D) for 21 consecutive days starting the day following the first iron-dextran injection. All experimental animals were sacrificed on 22^nd^ day under mild anesthesia (ethyl ether), and cardiac puncture was performed to collect blood and serum was separated and stored at −80 °C. After collecting the blood, the liver was quickly excised, cleaned thoroughly with cold phosphate buffer saline (PBS) to remove the remaining blood and cut into three sections. The major liver portion was dissected and homogenized using 10 volumes of 0.1 M phosphate buffer (pH 7.4) supplemented with 0.15 M NaCl and 5 mM EDTA and centrifuged for 30 min at 8000 g in the cold. The clear homogenate (supernatant) was collected and the protein concentration was quantified by Folin-Lowry method [11], where BSA was used as a standard; the remaining supernatant was then stored at −80 °C. Second liver fragment was treated with a mixture of nitric acid and sulfuric acid (1:1) to analyze the iron content. The last portion was processed for histopathological examinations.

#### Serum ferritin and liver iron levels

Manufacturer’s instructions were followed to quantify serum ferritin levels using an ELISA kit from Monobind Inc., USA. Liver iron content was quantified using a previously reported method [12]. Briefly, samples were mixed with bathophenanthroline sulfonate and incubated at 37 °C for 30 min, and absorbance was recorded using a spectrophotometer (Shimadzu, UV-2401PC, Japan) at 535 nm.

#### In vitro ferritin iron release

Iron reduction and release were determined using ferrozine, a spectrophotometric reagent for iron, as previously described [13]. Briefly, the reaction was initiated by adding different concentrations (100–500 μg/ml) of test compounds in 50 mM phosphate buffer (pH 7.0) containing 200 μg ferritin and 500 μM ferrozine, and the absorbance change was measured for 20 min at 560 nm. The reaction mixture excluding the test samples was used as a reference.

#### Serum markers

Aspartate amino transferase (ASAT), alanine amino transferase (ALAT), and bilirubin levels in serum were evaluated using commonly available kits from Merck, India. Similarly, alkaline phosphatase (ALP) levels were measured by a kit from Sentinel diagnostics, Italy.

#### Antioxidant enzymes

Superoxide dismutase (SOD), catalase (CAT), glutathione-S-transferase (GST), and reduced glutathione (GSH) levels were measured using previously described methods [14–17].

#### Measurement of ROS levels in liver, spleen homogenate and serum

ROS levels in liver and spleen tissue homogenate and serum were estimated using 2,7-dichlorofluorescein diacetate (DCFDA) following the method of Rashid et al. [18] with slight modifications. Briefly, the sample containing 50 μg protein was mixed with 1 ml assay buffer (130 mM KCL, 5 mM MgCl_2_, 20 mM NaH_2_PO_4_, 20 mM Tris HCl, 30 mM Glucose and 10 μM DCFDA) and incubated at 37 °C for 15 min in the dark. DCF formation was recorded at the excitation wavelength of 488 nm and emission wavelength of 523 nm for 2 min using a fluorescence spectrometer (Hitachi, Model F4500) that had been equipped with a FITC filter.

#### Evaluation of liver damage and fibrosis

Thiobarbituric acid reactive substances (TBARS) quantities were measured to evaluate the levels of lipid peroxidation [19]. Protein oxidation levels were resolved by estimating protein carbonyl contents [20]. Collagen content, an important marker of liver fibrosis, was evaluated using previously described methods [21]. Collagen content in each sample was determined by multiplying 7.69 by overall hydroxyproline content [22].

#### Histopathological studies

Excised liver samples were cleaned with saline and fixed for two days in 10 % buffered neutral formalin. Sections (5 μm thick) were paraffin-embedded and stained with hematoxylin and eosin (morphological examination), Perls’ Prussian blue dye (iron content) and Masson’s trichrome stain (liver fibrosis). Stained sections were checked microscopically for histopathological changes.

#### Statistical analysis

All of the data were reported as the mean ± SD of six measurements. Statistical analysis was performed using KyPlot version 2.0 beta 15 (32 bit) and Microsoft excel 2010. The relationships between the groups were evaluated using a paired *t*-test for in vitro study and one way ANOVA followed by Tukey's post hoc tests for in vivo study, and a *p* value of <0.05 was considered to be statistically significant.

## Results

### In vitro iron chelation and reducing power potential

**Fig. 1:**
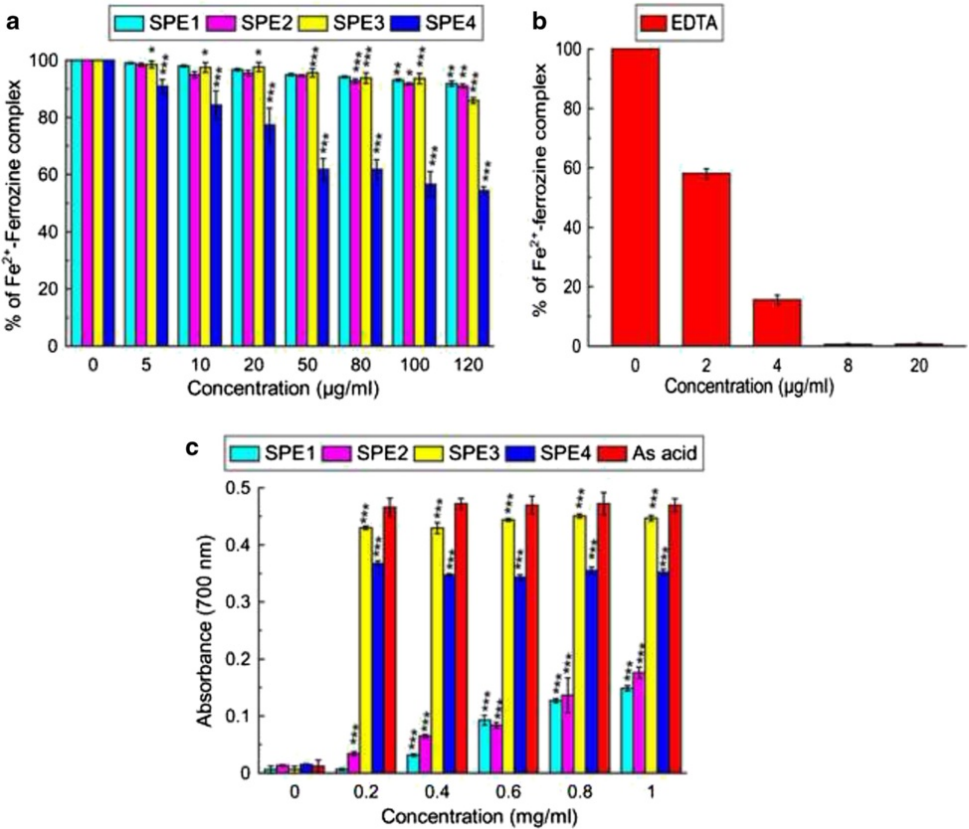
Iron chelation and reducing power activity of the isolated compounds. **a** iron chelation activity of the compounds, **b** Standard EDTA **c** Reducing power assay. ‘As acid’ represents Ascorbic Acid. The results represent the mean ± S.D. (*n* = 6). *p < 0.05, **p ≤ 0.01 and ***p < 0.001 vs. control

**Fig. 2:**
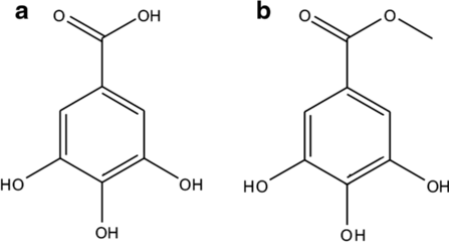
Chemical structure of the identified compounds. **a** Gallic acid and **b** Methyl gallate from *S. pinnata* bark

The iron chelation potential of the isolated compounds was measured using two essential methods: Fe^3+^ reducing power and iron chelation properties. SPE1 and SPE2 failed to show any activity in both the assays and SPE3, standard ascorbic acid demonstrated corresponding reducing power activities, which were although higher than SPE4 (Fig. 1a). Conversely, SPE4 demonstrated excellent and better in vitro iron chelation activity than SPE3 (Fig. 1b). The structure of SPE3, SPE4 (Fig. 2) was determined as Gallic acid (GA), and Methly gallate (MG) using different spectroscopic experimental data shown in Additional file 1. The IC_50_ value of MG and GA in iron chelation property is 129.85 ± 6.90 μg/ml and 1007.35 ± 141.31 μg/ml (*n* = 6) respectively.

### In vivo studies

#### Serum ferritin and liver iron levels

**Fig. 3:**
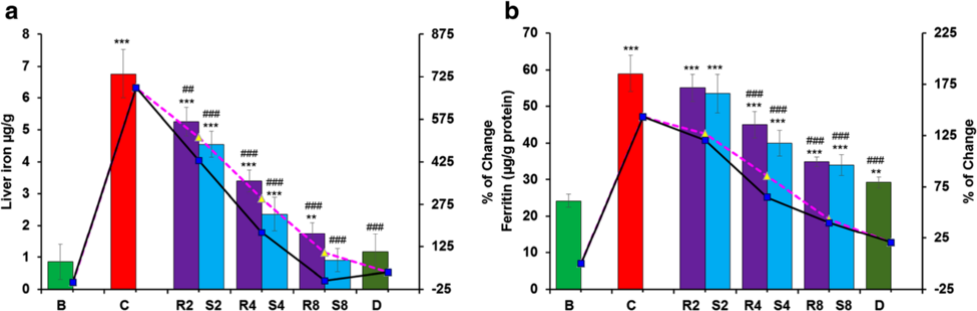
Iron removal potential of GA and MG. **a** Hepatic iron content, **b** Serum ferritin content. Mouse groups (B; C; R2; S2; R4; S4; R8; S8; D) were treated as described in the ‘Experimental design and tissue preparation’ section. Values are expressed as the mean ± SD of six mice. **p ≤ 0.01, ***p ≤ 0.001 compared with the blank and ^###^ *p* ≤ 0.001 compared with the control

Increase in hepatic iron content and serum ferritin levels were observed in the iron-overloaded mice compared with the normal mouse group. Upon GA and MG administration, notable tendencies of a dose-dependent reduction in liver iron content (Fig. 3a) as well as serum ferritin concentrations (Fig. 3b) were observed. The highest dose of MG demonstrated better activity than the standard drug desirox and significantly eliminated the excess iron, thus making the condition equal to the normal mice group.

#### Iron release from ferritin

**Fig. 4:**
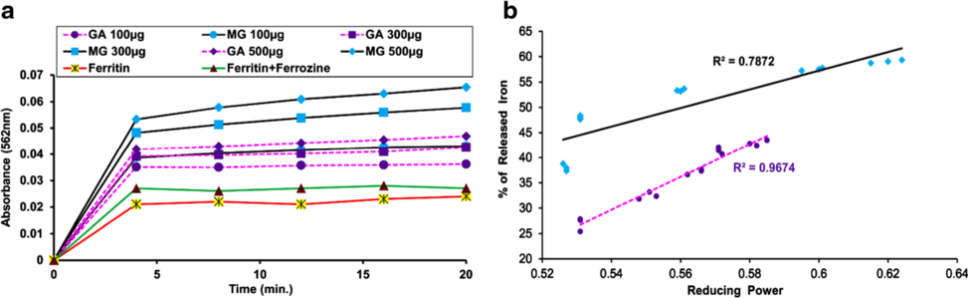
Iron release from ferritin. **a** Iron release from ferritin **b** Correlation between iron released from ferritin with reducing power. Iron released in response to increasing amounts (100–500 μg) of both GA and MG was plotted against reducing power displayed at the same doses

A dose dependent increase in the formation of the ferrous-ferrozine complex [(Fe(ferrozine)3)^2+^] was measured to quantify the efficiency of GA and MG in releasing the reduced iron from ferritin. Experiments without test samples formed insignificant quantities of the reduced iron ferrozine complex, whereas increasing concentrations significantly released iron from ferrozine with time (Fig. 4a). MG also demonstrated better activity than GA, but when the percentage of iron released from ferritin and the reducing power of the test samples were correlated (Fig. 4b).

#### Antioxidant enzymes

**Fig. 5:**
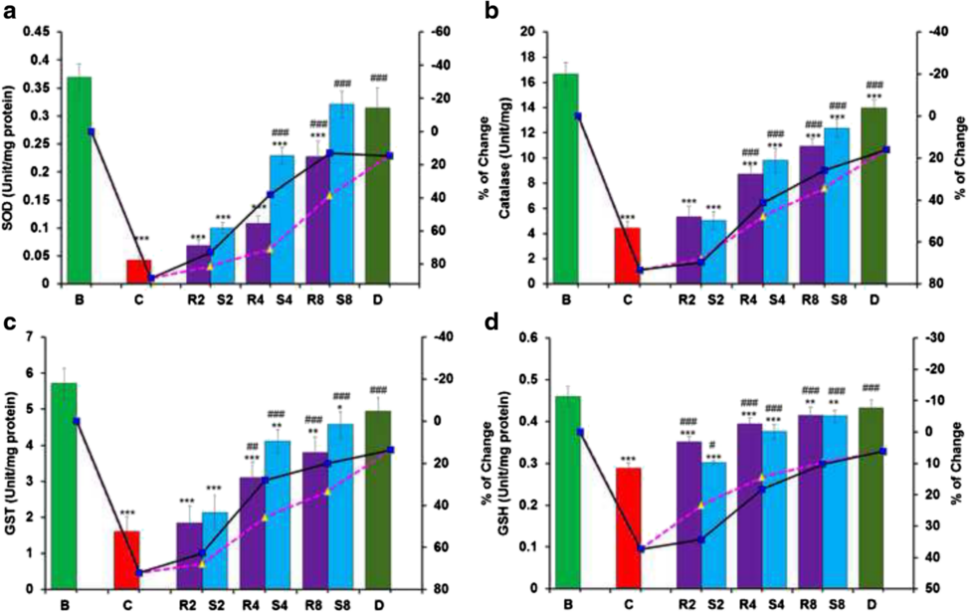
Effect of GA and MG treatment on liver antioxidant enzyme levels. **a** SOD, **b** Catalase, **c** GST, **d** GSH. Mouse groups (B; C; R2; S2; R4; S4; R8; S8; D) were treated as described in the ‘Experimental design and tissue preparation’ section. Values are expressed as the mean ± SD (*n* = 6). *p < 0.05, **p ≤ 0.01, ***p ≤ 0.001 compared with the blank and ^#^ *p* ≤ 0.05, ^##^ *p* ≤ 0.01, ^###^ *p* ≤ 0.001 compared with the control

The iron-overloaded condition stimulated an oxidative stress condition that significantly reduces levels of antioxidant enzymes and non-enzymatic antioxidant GSH compared with normal mice. Oral administration of GA and MG indicated significant restoration of the antioxidant enzymes almost to the normal state. In the case of SOD (Fig. 5a), MG exhibited better activity than standard treatment. At the highest MG dose, catalase (Fig. 5b), GST (Fig. 5c) and GSH (Fig. 5d) levels nearly approached standard desirox levels, while GA displayed moderate activity.

#### ROS levels

**Fig. 6:**
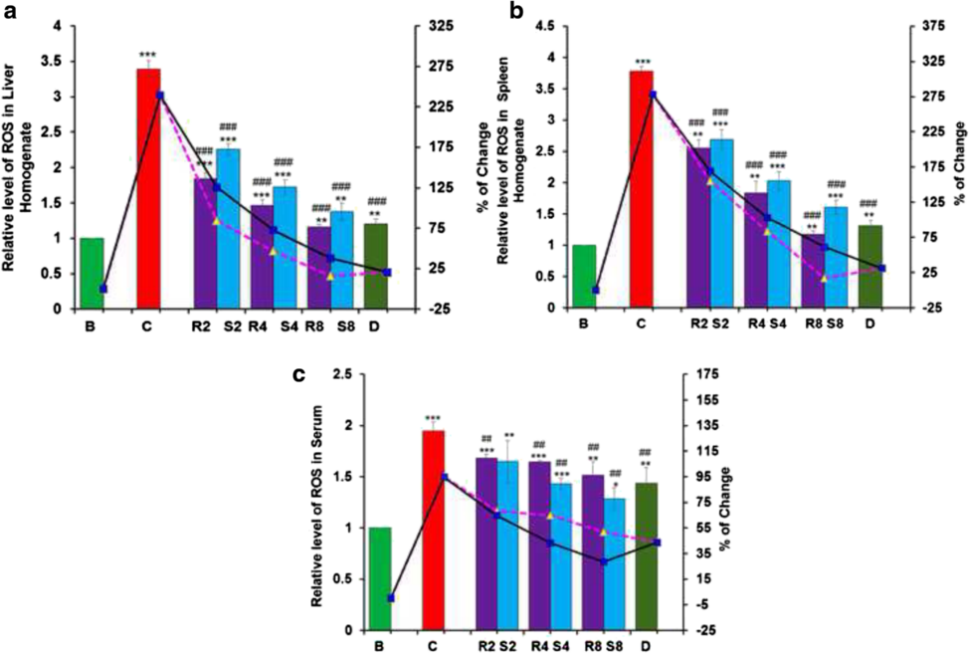
Restoration of ROS levels in liver, spleen and serum after GA and MG treatment. **a** Relative ROS levels in liver homogenates, **b** Relative ROS levels in spleen homogenates **c**. Relative ROS levels in serum. Mouse groups (B; C;R2; S2; R4; S4; R8; S8; **d** were treated as described in the ‘Experimental design and tissue preparation’ section. Values are expressed as the mean ± SD (*n* = 6). *p < 0.05, **p ≤ 0.01, ***p ≤ 0.001 compared with the blank and ^##^ *p* ≤ 0.01, ^###^ *p* ≤ 0.001 compared with the control

The ROS levels in liver and spleen homogenates as well as serum were increased in the iron overloaded mice compared with normal mice. Upon GA and MG administration, the levels gradually regressed towards the normal condition (Fig. 6a, b and c, respectively). Treatment with GA reduced the levels of ROS in liver and spleen homogenate better than MG as well as desirox treatment. But serum ROS levels were more greatly reduced by MG than GA treatment.

#### Serum markers

The elevated levels of serum enzymes and bilirubin markedly dose-dependently decreased after oral GA and MG administration. From Table 1, it is clear that higher doses of MG displayed better activity than GA and the standard desirox in all cases except for bilirubin.

**Table 1:** The effect of GA and MG on serum marker enzymes (ALAT, ASAT and ALP) and Bilirubin in iron overloaded mice

Treatment	ALAT (Unit/L)		ASAT (Unit/L)		ALP (Unit/L)		Bilirubin (mg/dl)	
	GA	MG	GA	MG	GA	MG	GA	MG
B	14.42 ± 1.91	14.42 ± 1.91	62.46 ± 6.32	62.46 ± 6.32	84.62 ± 3.48	84.62 ± 3.48	1.58 ± 0.10	1.58 ± 0.10
C	64.60 ± 4.32 ^X3^	64.60 ± 4.32 ^X3^	234.91 ± 8.30 ^X3^	234.91 ± 8.30 ^X3^	252.63 ± 4.92 ^X3^	252.63 ± 4.92 ^X3^	3.15 ± 0.18 ^X3^	3.15 ± 0.18 ^X3^
2 mg/kg b.w	42.96 ± 1.16 ^X3Y3^	36.93 ± 0.93 ^X3Y3^	145.45 ± 5.89 ^X3Y3^	141.51 ± 11.57^X3Y3^	181.90 ± 4.95 ^X3Y3^	173.41 ± 2.93 ^X3Y3^	2.88 ± 0.39 ^X3^	3.04 ± 0.25 ^X3^
4 mg/kg b.w	32.17 ± 2.16 ^X3Y3^	29.09 ± 0.59 ^X3Y3^	114.78 ± 8.20 ^X3Y3^	110.28 ± 6.55 ^X3Y3^	145.10 ± 6.46 ^X3Y3^	132.32 ± 3.56 ^X3Y3^	2.47 ± 0.24 ^X3Y3^	2.11 ± 0.16 ^X3Y3^
8 mg/kg b.w	23.22 ± 0.95 ^X3Y3^	20.25 ± 0.90 ^X2Y3^	83.56 ± 4.86 ^X3Y3^	77.93 ± 13.48 ^X1Y3^	118.69 ± 3.79 ^X3Y3^	103.34 ± 5.88 ^X3Y3^	2.10 ± 0.12 ^X3Y3^	1.94 ± 0.13 ^X3Y3^
D	19.85 ± 0.79 ^X3Y3^	19.85 ± 0.79 ^X3Y3^	88.06 ± 10.37 ^X3Y3^	88.06 ± 10.37 ^X3Y3^	114.97 ± 6.13 ^X3Y3^	114.97 ± 6.13 ^X3Y3^	1.72 ± 0.14 ^Y3^	1.72 ± 0.14 ^Y3^

Values are mean ± SD of six observations. Treatment groups are B: Normal mice; C: Iron-dextran treated mice receiving normal saline; 2 mg/kg b.w. treated group; 4 mg/kg b.w. treated group; 8 mg/kg b.w. treated group; D: 20 mg/kg b.w. desirox treated group. X1: *p <* 0.05, *X*2: *p* < 0.01 and X3: *p <* 0.001 significant difference from normal mice (B) group. Y1: *p <* 0.05, Y2: *p <* 0.01 and Y3: *p <* 0.001 significant difference from iron overloaded (C) group

#### Biochemical parameters of liver damage

**Fig. 7:**
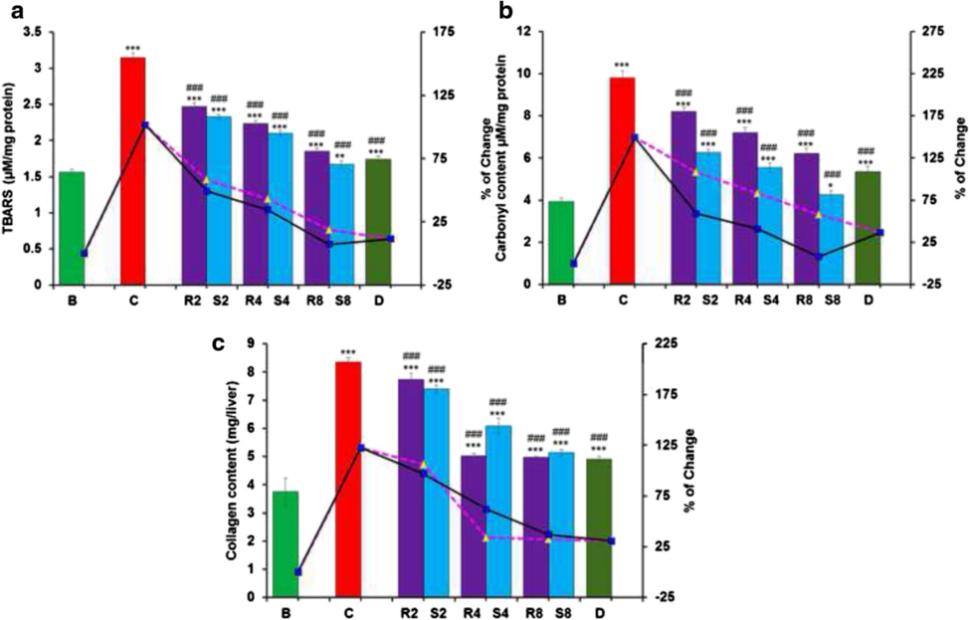
Effect of GA and MG treatment on liver damage parameters. **a** Lipid peroxidation levels, **b** Protein carbonyl content, **c** Collagen content. Mouse groups (B; C; R2; S2; R4; S4; R8; S8; **d** were treated as described in ‘Experimental design and tissue preparation’ section. Values are expressed as the mean ± SD (*n* = 6). *p < 0.05, **p ≤ 0.01, ***p ≤ 0.001 compared with the blank and ^###^ *p* ≤ 0.001 compared with the control

The effect of iron overload on liver damage was measured by the level of lipid peroxidation, protein carbonyl and collagen content in the liver homogenate. When treated with the test compounds, thiobarbituric acid reactive substance (TBARS) levels, which measures MDA, were substantially decreased (Fig. 7a). In case of protein carbonyl content, MG exceptionally prevented protein oxidation because protein carbonyl content is almost similar to normal mice (Fig. 7b). However, in the case of hydroxylproline, i.e., collagen content, GA exhibited marginally better activity than MG and similar activity to standard desirox (Fig. 7c).

#### Histopathological study

**Fig. 8:**
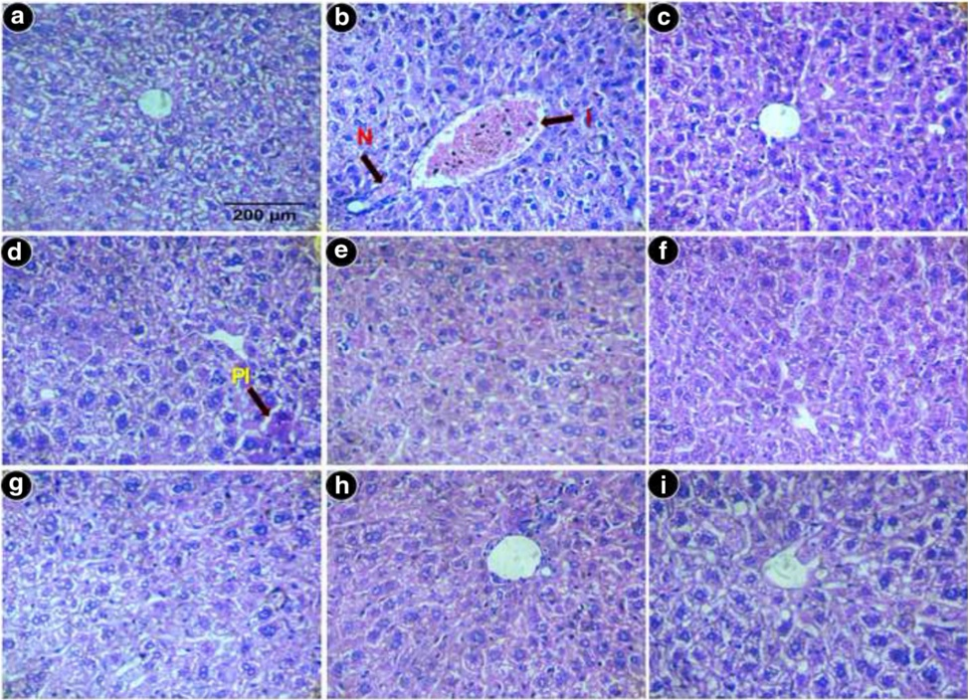
Microscopic observation of mouse liver sections that had been stained with hematoxylin and eosin at × 400. Liver sections from control mice with normal cytoarchitecture. **b** Iron-overloaded (iron dextran, 100 mg/kg b.w.) liver section demonstrates degeneration of cellular boundaries, fatty ballooning deterioration, inflammation (I), and necrosis (N). **c** Desirox-treated liver sections demonstrate reduced necrotic area. **d** Liver section from the R2 mouse group improved histology with portal inflammation (PI). **e** Liver section from the R4 mouse group (**f)** Liver section from the R8 mouse group (**g)**. Liver section from the S2 mouse group (**h**). Liver section from the S4 mouse group (**i**). Liver section from the S8 mouse group demonstrates improved histology with minimal necrotic area

**Fig. 9:**
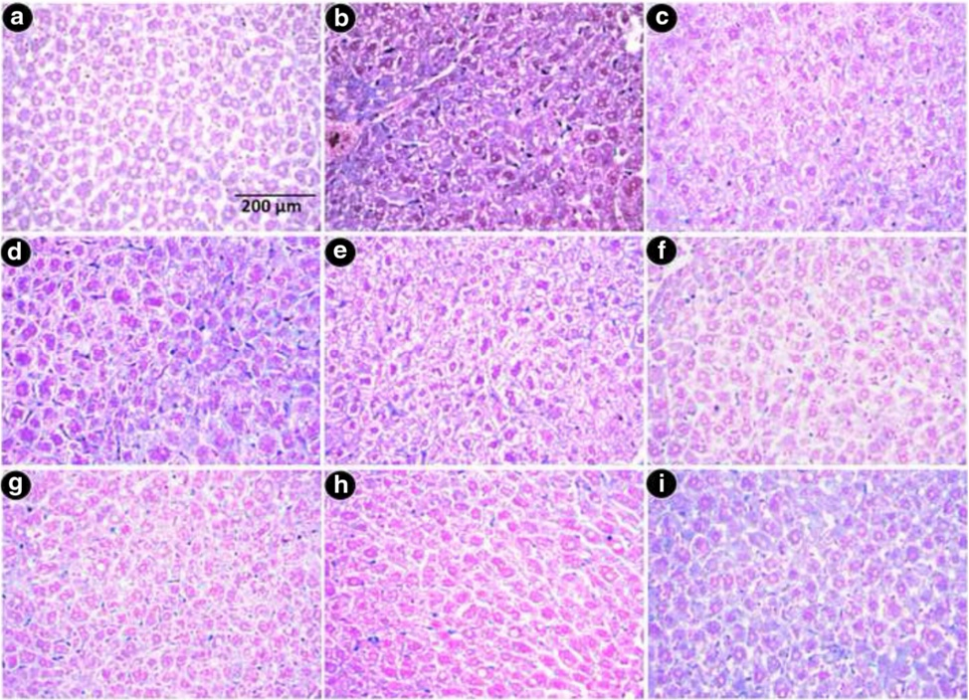
Microscopic observation of liver sections stained with Perls’ Prussian blue at × 400. **a** Liver section from control mice demonstrates normal hemosiderin deposition patches (very low). **b** Liver sections from iron-overloaded mice display excess blue patches. **c** Desirox-treated liver section **d** Liver section from the R2 mouse group with lesser blue patches. **e** Liver section from the R4 mouse group (**f**). Liver section from the R8 mouse group (**g**). Liver section from the S2 mouse group (**h**). Liver section from the S4 mouse group (**i**). Liver section from the S8 mouse group. MG demonstrates improved histology, and a gradual reduction of blue patches indicates effective iron removal from the liver

**Fig. 10:**
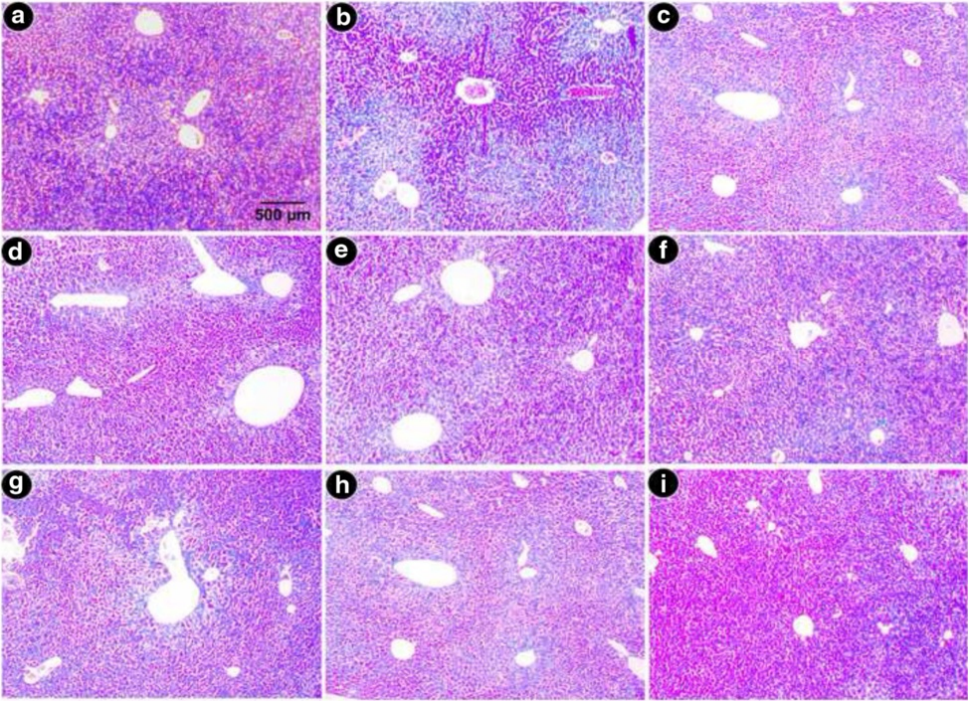
Microscopic observation of liver sections stained with Masson’s Trichrome × 100. **a** Liver sections from control mice displayed normal cellular integrity with no fibrosis. **b** Liver sections from iron-overloaded mice displayed elongated fibrous septa and collagen accumulation (*blue*). **c** Desirox-treated liver section **d**. Liver section from the R2 mouse group with lesser blue patches. **e** Liver section from the R4 mouse group (**f**). Liver section from the R8 mouse group (**g**). Liver section from the S2 mouse group (**h**). Liver section from the S4 mouse group (**i**). Liver section from the S8 mouse group. MG demonstrates a nearly negligible collagen accumulation and healthy liver. Higher doses of both GA and MG demonstrate reduced collagen deposition, fibrous septum and necrotic cells in periportal veins, indicating a trend of restoration of normal cellular integrity

Biopsy sections were stained with hematoxylin and eosin for morphologic evaluation, Perls’ Prussian Blue stain for assessment of iron loading, and Masson’s trichrome stain for assessment of fibrosis. Liver sections from normal mice demonstrated normal cell morphology with prominent nuclei in well-preserved cytoplasm and prominent central vein without cellular infiltration (Fig. 8a), whereas iron dextran overloaded mice demonstrated various degrees of pathological changes including ballooning degeneration, inflammation, loss of cellular boundaries, and hepatocellular necrosis (Fig. 8b). In contrast, the liver sections taken from GA (Fig. 8d-f) and MG (Fig. 8g-i) treated mice groups displayed attenuation of pathogenicity and gradual reversal to normal cyto-architecture with a higher dosage, thus restoring the normal condition. Figure 8c represents liver sections of the desirox-treated group with improved histology, which is similar to the highest doses of GA and MG. Another detrimental effect of excess iron in liver is deposition of iron in the form crystalline ferritine and amorphous hemosiderin. Iron released from denatured ferritin, ferric oxide (unused iron) as well as broken hemoglobin formed a complex to store the iron known as hemosiderin. The iron within the deposits of hemosiderin is poorly available to the body and tissue sections stained with Perls’ Prussian blue is commonly used to detect its deposition in liver tissue as blue patches. The liver sections from untreated iron overloaded mice demonstrated increased hemosiderin deposition (Fig. 9b) compared with normal mice (Fig. 9a). However, sections from the treated mice groups demonstrated a gradual decrease in hemosiderin deposition patches (Fig. 9d-f for GA and 9g-i for MG). The highest dose of MG exhibited a parallel effect to the standard desirox-treated group (Fig. 9c). Accumulated collagen in liver was also stained blue using Masson’s trichrome. The microscopic observation suggested that the liver section of control mice revealed normal lobular architecture and distribution of collagen (Fig. 10a). From the liver section of iron-overloaded mice it is evident that the normal architecture of the liver is destroyed and the nodules surrounded by accumulated collagen indicating fibrous cirrhotic (Fig. 10b). However, after treatment with the test samples, a gradual decrease in the degree of collagen deposition was observed (Fig. 10d-f for GA and 10g-i for MG). Here, the highest MG dose also demonstrated a similar scenario compared with the standard desirox-treated group (Fig. 10c).

## Discussion

Free radicals and iron metal are essential entities of aerobic life and modulate various physiological functions [23]. In iron-overloaded liver, iron reacts with the cellular hydrogen peroxide to generate hydroxyl radical (Fenton reaction) which in turn initiate the propagation of various ROS. This situation leads to oxidative stress which damage in liver tissue mostly via lipid peroxidation of biological membranes [24]. However, the preventive system of our body is inadequate to handle excess free radicals; therefore, external antioxidant iron chelator supplements are essential to maintain healthy physiological conditions. An effective remedial strategy should act in a dual character by decreasing the oxidation rate; one method is sequestering and chelating the stored iron in cells [25], and the other is a radical trap (i.e., antioxidant activity). Though the development of some synthetic antioxidants and iron chelating drugs has thrived recently, they are not yet widely accepted as therapeutic agents due to several side effects and disadvantages. Thus, there is a growing demand to develop more effective and safe therapeutic approaches to chelate catalytically active cytosolic iron [25] and concurrently protect cells from excess free radicals. Several bio resources have already been reported as natural antioxidants with iron chelating potentials [26, 27].

Results of in vitro iron chelation and reducing power property of the isolated compound from ethyl acetate fraction suggest that on SPE3 and SPE4 do possessed both the activities where other two compounds failed to show any activity. So, the structure of SPE3 and SPE4 was elucidated and found to be gallic acid (GA) and methyl gallate (MG). MG exhibited better iron chelation than GA although their basic structure is alike. So, this iron chelation result further supported the findings of Yang et al. [28] who suggested that Fe(III) in Hepes buffer, pH 7.4 interacted with three molecules of MG and produced a Fe(III)–MG_3_ complex with a stable octahedral geometry (binding constant- K is in the range of 1 × 10^34^/M to 1 × 10^36^/M) and chelate-free iron better than GA.

Both the compounds, GA and MG have demonstrated excellent in vitro iron chelation anf reducing power potency, a study on the in vivo ameliorating effect of GA and MG in iron overloaded liver toxicity and liver fibrosis in mice was constructed. Hemochromatosis was created by intraperitoneal injection of iron-dextran. This process will not hamper intestinal iron absorption by the test compounds, which ultimately leads to iron overload in liver and serum [29].

The excess iron stored in the liver as ferritin or hemosiderin [30]. Thus, most procedures seek to measure liver iron levels for diagnosis of iron overload disease. In contrast, ferritin, a ubiquitous intracellular iron-binding protein, generally stores iron in a non-toxic ferric form and releases it in a controlled fashion whenever needed [31]. Serum ferritin is a key marker that was developed as a consequence of iron overload-induced hepatic toxicity, as blood ferritin content indirectly reveals the amount of hepatic iron content. The decrease in hepatic iron deposition after MG treatment justified its in vitro iron-chelating effectiveness.

In hepatocytes, ferritin stores excess iron in the ferric state. To overcome iron overload, various readily available iron chelator drugs are administered, but many of them struggle with a narrow binding capacity for ferric iron (Fe^3+^). Thus, reducing agents such as ascorbic acid need to be administered as supplements to upsurge the accessibility of stored iron to chelators [32]. From the result it was found that both MG and GA effectively release iron from ferritin which also nicely correlated with their reducing power capacity. GA possessed a greater correlation coefficient (*R*^2^ = 0.9674) than MG (*R*^2^ = 0.7872). This also validated the superior reducing power activity of GA over MG.

Living cells are equipped with an array of endogenous antioxidant enzymes such as GST, SOD, CAT and the small compound GSH, which are first line of defenses against excessive free radicals. Evaluating the levels of these antioxidant enzymes is a proper indirect way to assess pro-oxidant-antioxidant combat in tissues [33]. SOD destroys superoxide free radicals by converting them into H_2_O_2,_ whereas catalase further decomposes excess H_2_O_2_ to H_2_O and O_2_. Catalase efficiency is so commendable that it cannot be saturated by any concentration of H_2_O_2_ [34]. The cellular GSH system is probably the most important cellular defense mechanism, which not only acts as a ROS scavenger but also regulates the intracellular redox state [35]. The results suggested that MG restore the antioxidant enzymes much better than GA and the activity is almost equal to standard drug Desirox (20 mg/kg.b.w) but in lower concentration (8 mg/kg.b.w).

The previous phenomenon can also be further supported by the obtained ROS levels in liver spleen homogenates and serum in iron overloaded condition as well as after treatment. Surprisingly, GA exhibited much better activity than MG and desirox, which also supported its excellent antioxidant and free radical scavenging potentials. On the other hand, MG exhibited better ROS level reduction in serum of the iron overloaded mice. It is speculated that MG, which is an excellent iron chelator, assists albumin to further chelate excess iron, thereby lowering serum ROS levels. Generally, albumin represents the predominant antioxidant in plasma, which is exposed to continuous oxidative stress [36]. Albumin concentrations increase in inflammation, and the antioxidant activities of albumin result from its ligand-binding capacities of ROS-producing metal such as copper and iron [37].

Levels of serum enzyme are checked in the clinical diagnosis to determine the condition of various diseases and tissue injury [38, 39]. These enzymes are predominantly found in the hepatic cell and liver damage due to excess iron leads to the release of these intracellular enzymes into the blood [40] as evident by increased serum parameters (Table 1). The administration of the test compounds has reduced the increased level of the serum marker enzymes almost similar to the standard desirox. This result indicated towards their healing capabilities of hepatic parenchyma and regeneration of hepatocyte as well as its functional efficiency [41]. Hence, MG not only helped to overcome oxidative stress but also possessed better hepato-amelioration activity compared with GA.

Cell membrane lipid peroxidation is the most detrimental result of iron-induced oxidative stress mainly via hydroxyl radicals [42]. Lipid peroxidation (LPO) and the resulting end products such as malondialdehyde (MDA) act as an important factors of liver fibrosis by activating hepatic stellate cells, resulting in increased pro-collagen α_1_ (I) gene expression [43–45]. This relationship between lipid peroxidation and hepatic fibrosis is well established in a variety of liver diseases including hemochromatosis, alcohol-induced liver injury and chronic hepatitis C [46]. Subsequent to hepatic cellular damage, collagen production predominates over hepatocellular regeneration as an immediate healing response, thereby occupying the injured areas instead of destroyed hepatocytes. Collagen content is therefore considered to be a major marker of liver fibrosis and hepatotoxicity [47]. Iron-induced liver pathogenicity also leads to the oxidation of various important structural and functional proteins and forms protein carbonyls. Thus, it serves as a marker of oxidative stress and leads to the onset/development of various diseases including cystic fibrosis and ulcerative colitis [30]. The chelation of excess iron by MG and GA further strengthen the position of GA and MG in significantly overcoming hepatic damage/fibrosis in iron intoxicated mice, indicating the hepato-ameliorating potency of the compounds.

A liver biopsy is considered to be the gold-standard method for assessment of the degree of inflammation and fibrosis alongside other biochemical tests. The liver sections stained with hematoxylin and eosin exhibited various degrees of inflammation, necrosis as well as cell wall degeneration in iron overloaded condition, but dose dependent treatment reduced the tissue damage that also supported the result of the restoration of serum enzyme and antioxidant enzyme levels. Perls’ Prussian blue staining revealed the visual confirmation of iron chelation property of the test compounds along with the liver iron content and serum ferritin content. On the other hand, Masson’s trichrome staining disclosed the reduction of liver fibrosis as the collagen content gradually decreased with the increasing doses of GA and MG, which also confirmed the anti-fibrotic effect of the fraction along with the test for collagen content. Overall results in histopathological studies signifying in situ evidence of ameliorating the effect of the iron overload-induced liver toxicity.

## Conclusion

Excess iron plays a critical role in oxidative stress-related hepato-toxicity and liver fibrosis, which is profoundly supported by the results of the present study. Among the isolated compounds from *S. pinnata* bark, MG exhibited exceptional amelioration potential for hepatotoxicity mainly due to its iron chelation capacity. In contrast, GA failed to demonstrate brilliant iron chelation activity in vitro, but it displayed a pleasing amendment of oxidative stress and liver damage as it neutralized excess generated free radicals due to iron overload before they could attack any bio-entities. Thus, these two compounds act in different pathways to achieve the same goal. Although the isolated compounds showed similar effect as desirox, they proved the rationale behind the activity of the *S. pinnata* bark extract, which was already proven as better and safer drug than standard Desirox. These findings also suggest the beneficiary role of GA and MG in the pathological sequence of iron-overload-linked liver disease and fibrosis.

## Abbreviations

ALAT, alanine aminotransferase; ALP, alkaline phosphatase; ANOVA, analysis of variance; ASAT, aspartate aminotransferase; b.w., body weight; BSA, bovine serum albumin; CAT, catalase; CDNB, 1-chloro-2,4-dinitrobenzene; CNCI, Chittaranjan National Cancer Institute; CPCSEA, Committee for the Purpose of Control and Supervision of Experiments on Animals; CRIA, Central Research Institute of Ayurveda; DCFDA, 2,7-dichlorofluorescein diacetate; DTNB, 5,5′-dithiobis-2-nitrobenzoic acid; EDTA, ethylenediamine tetraacetic acid; EIMS, electron ionization mass spectrometry; ELISA, enzyme-linked immunosorbent assay; FITC, fluorescein isothiocyanate; FT-IR, fourier transform infrared spectroscopy; g, gram; GA, gallic acid; GSH, reduced glutathione; GST, glutathione-S-transferase; HPLC, high performance liquid chromatography; i.p, intraperitoneal; kg, kilogram; M, molar; MDA, malondialdehyde; MG, methyl gallate; mg, milligram; ml, milliliter; mM, Millimolar; NED, N-(1-Naphthyl) ethylenediamine dihydrochloride; NMR, nuclear magnetic resonance; PBS, phosphate buffer saline; RNS, reactive nitrogen species; ROS, reactive oxygen species; SD, standard deviation; SOD, superoxide dismutase; TBARS, thiobarbituric acid reactive substances

## Additional file

Additional file 1: Spectroscopic data of SPE3 and SPE4. (DOCX 9715 kb)
