# Supplementary material for: Role of phenolics from Spondias pinnata bark in amelioration of iron overload induced hepatic damage in Swiss albino mice
Source: BMC Pharmacol Toxicol. 2016 Jul 26;17:34. doi: 10.1186/s40360-016-0077-6 (PMC4962386; doi:10.1186/s40360-016-0077-6)
Supplement: Additional file 1: — Spectroscopic data of SPE3 and SPE4. (DOCX 9715 kb) [file 40360_2016_77_MOESM1_ESM.docx]

Supplementary Information For:

**Role of phenolics from *Spondias pinnata* bark in amelioration of iron overload induced hepatic damage in Swiss albino mice**

Dipankar Chaudhuri^#^, Nikhil Baban Ghate^#^, Sourav Panja, Nripendranath Mandal*

Division of Molecular Medicine, Bose Institute, P 1/12, C. I. T. Road, Scheme – VIIM, Kolkata - 700054, West Bengal, India

**#Authors contributed equally**

**Correspondence and reprint requests:**

Nripendranath Mandal

Division of Molecular Medicine, Bose Institute, P 1/12, C. I. T. Road, Scheme - VIIM, Kolkata - 700054, West Bengal, India

Tel: +91-33-2569-3285, Fax: +91-33-2355-3886

E-mail: mandaln@rediffmail.com


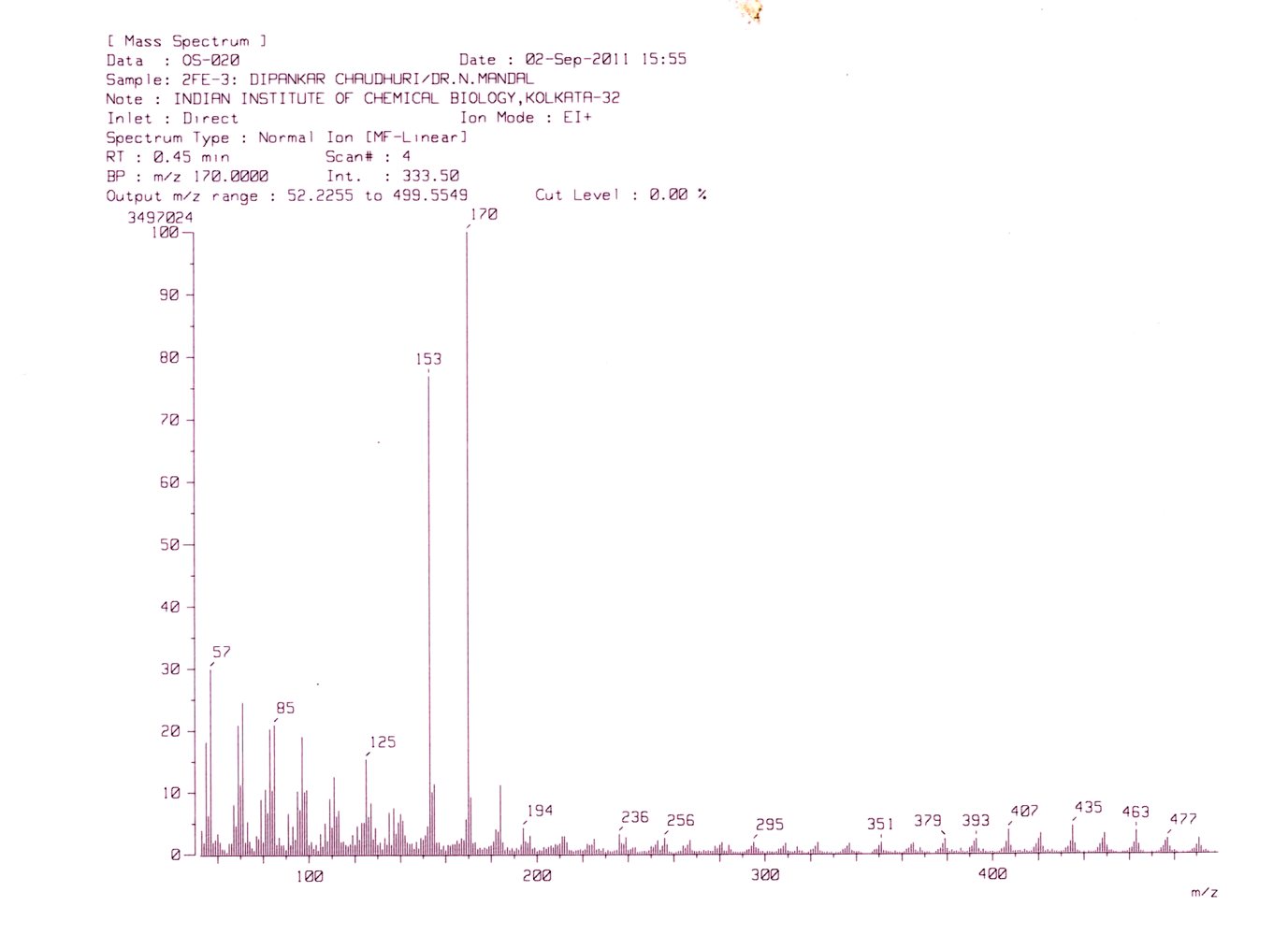


Fig. S1. High resolution mass spectra for SPE3.


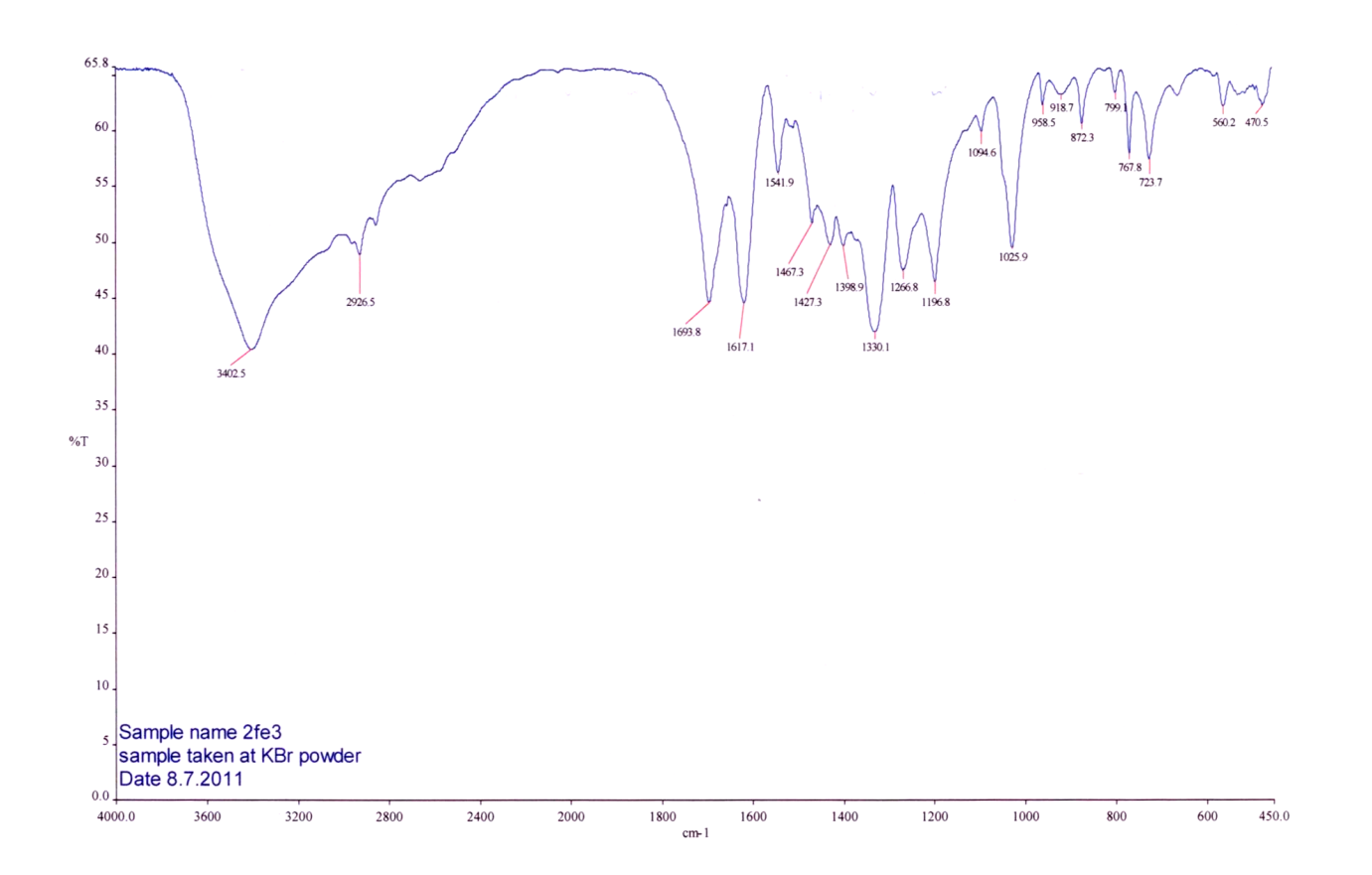


Fig. S2. FTIR spectra of SPE3


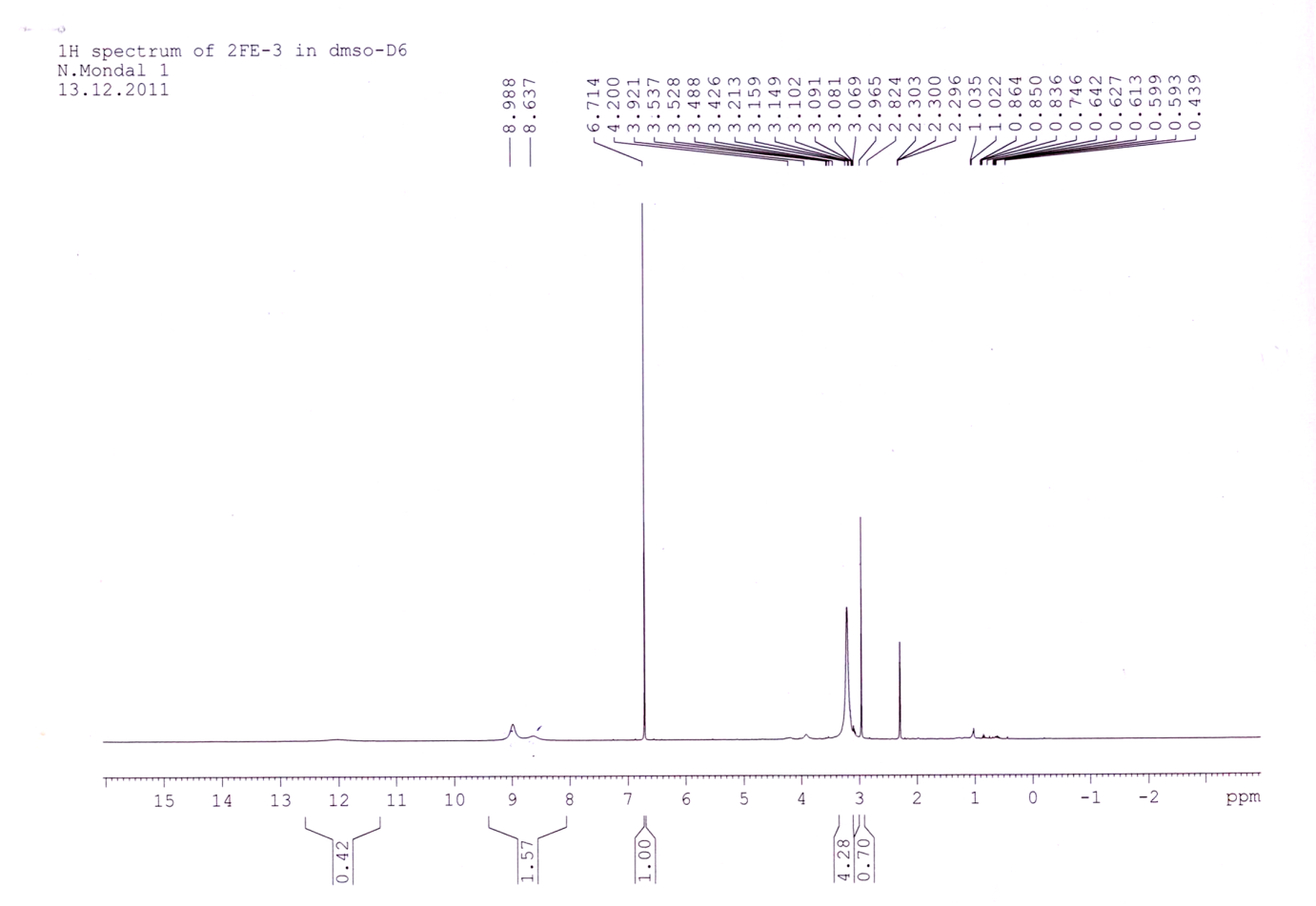


Fig. S3. 1H NMR spectra of SPE3


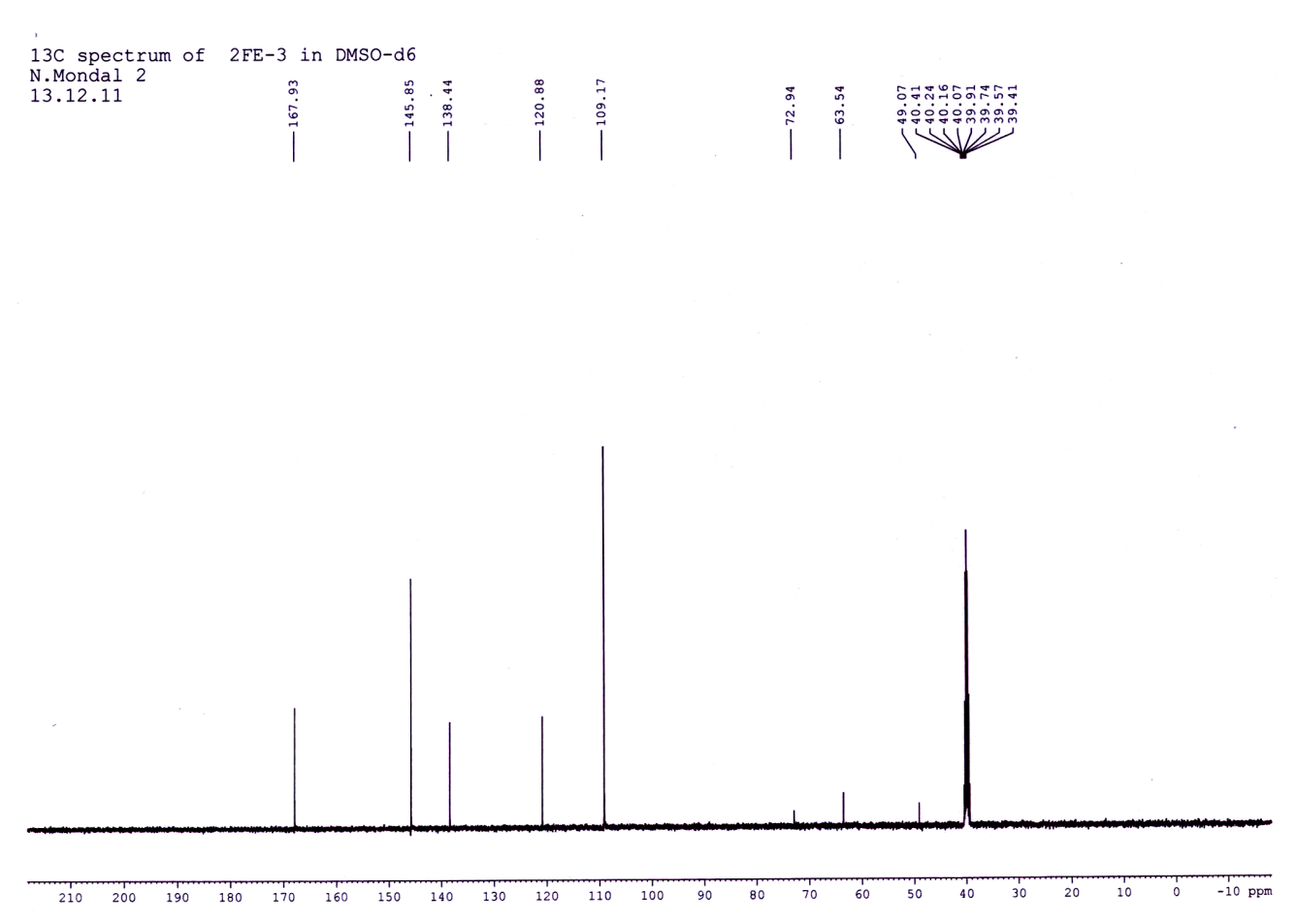


Fig. S4. 13C NMR spectra of SPE3


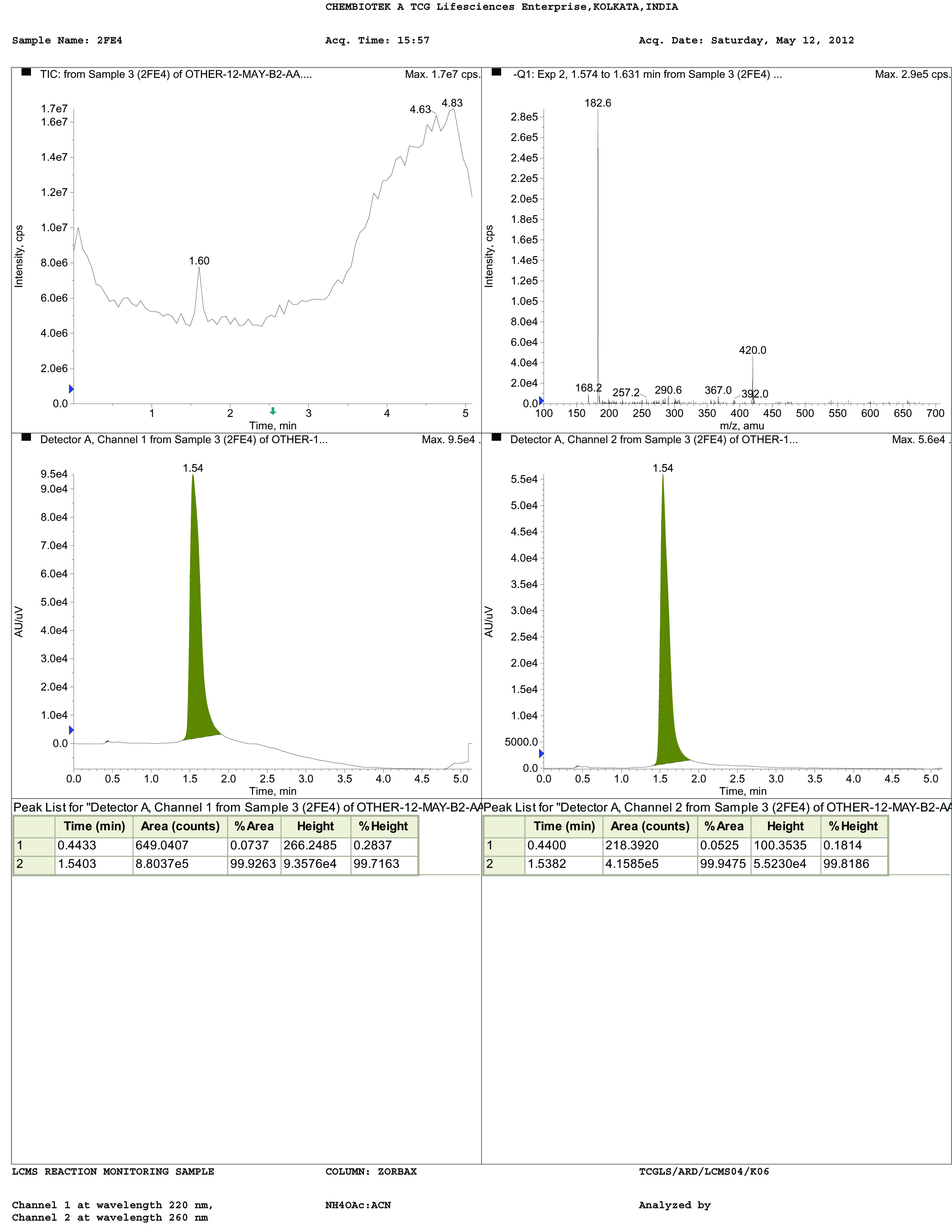


Fig. S5. Liquid chromatography mass spectra for SPE4


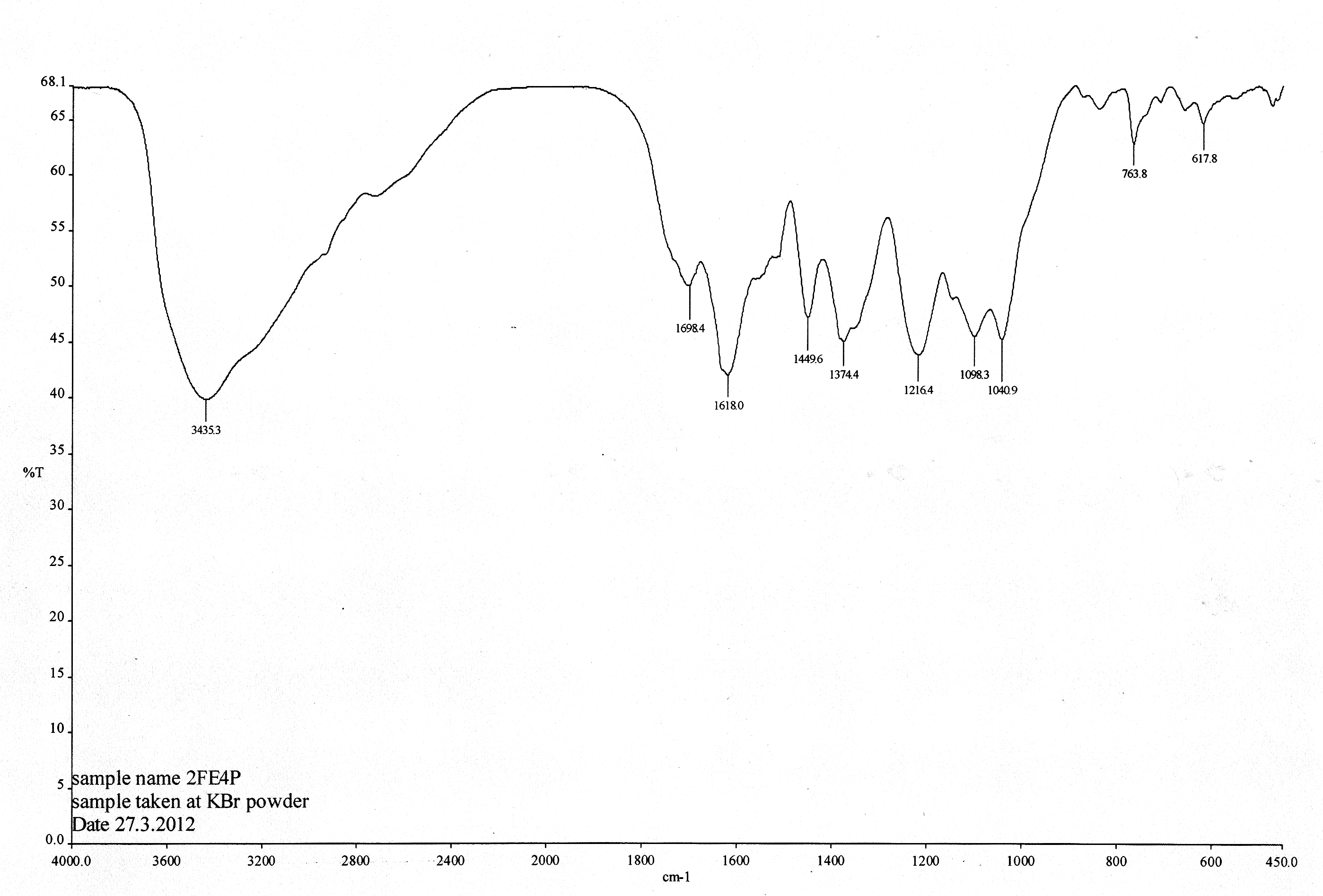


Fig. S6. FTIR spectra of SPE4


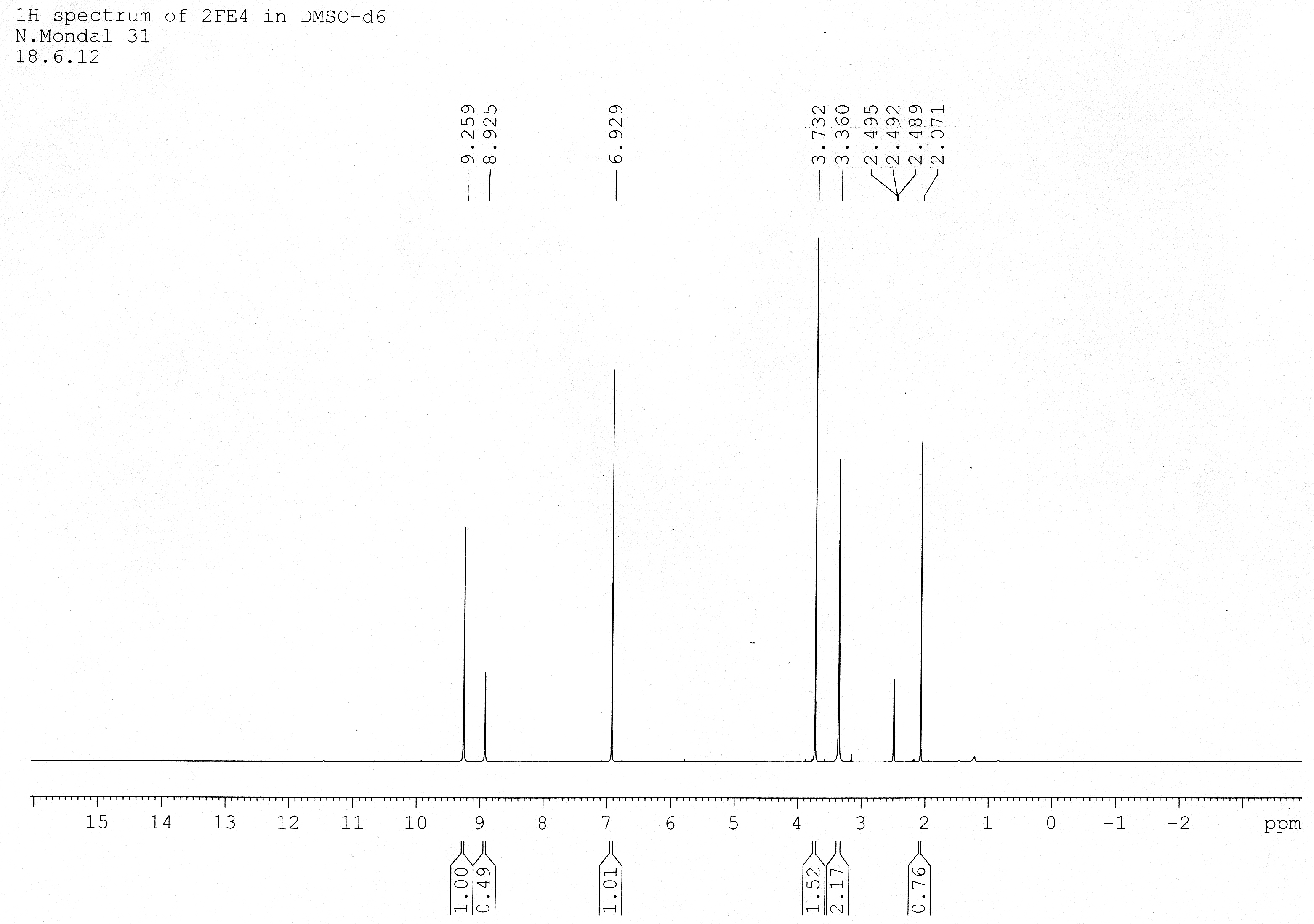


Fig. S7. 1H NMR spectra of SPE4


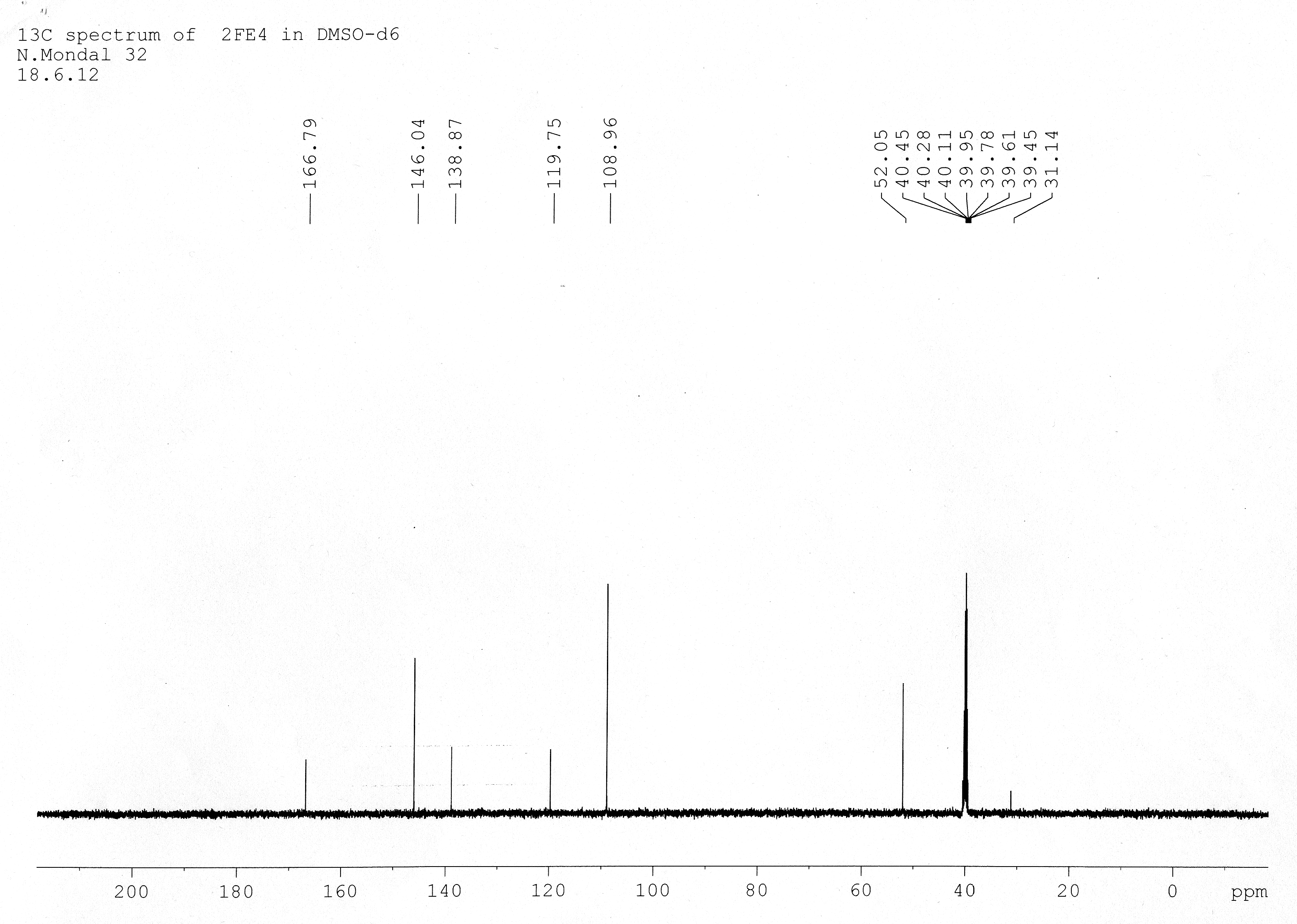


Fig. S8. 13C NMR spectra of SPE4
